# Analysis of cytokine profile and heme metabolism byproducts after hemorrhagic stroke

**DOI:** 10.1186/cc12647

**Published:** 2013-06-19

**Authors:** CR Shinotsuka, FA Bozza

**Affiliations:** 1 Hospital Copa Dor, Copacabana, Rio de Janeiro, RJ, Brazil

## Introduction

Intracerebral hemorrhage is a fatal disease, accounting for about 15% of deaths from stroke. Local and systemic inflammatory response associated with hemoglobin byproducts seems to have a causal role in neuronal death and ICH prognosis. The objective of this study was to investigate the relationship of cytokine profile and hemoglobin degradation products with brain injury severity and prognosis.

## Methods

We developed a prospective cohort study conducted in three tertiary hospitals. All ICH patients with hemoventricle and external ventricular device (EVD) inserted who were admitted to the neurocritical care unit between 2008 and 2011were included. We collected blood and cerebrospinal fluid (CSF) from the EVD on days 1, 2, 3, 5, and 7 after ICH for measurement of C-reactive protein, cytokines, heme, hemoglobin, cytometry, hemopexine, haptoglobin, enolase, and s-100B concentration. A multiplex analysis was performed to evaluate levels of 17 cytokines. CT scans were evaluated for hematoma and hemoventricle volume. Primary outcome was death in 7 days. This study was approved by the ethics committee of all participating institutions.

## Results

Fifteen patients were included. Median age was 59 years (IQR: 55 to 65), six patients (40%) were male. Median Glasgow Coma Scale was 7 (6 to 9), APACHE II score was 22 (IQR: 15 to 25) and SAPS III was 43 (IQR: 32 to 53). Five patients had hemorrhagic stroke and 10 subarachnoid hemorrhage. Overall mortality in 7 days was 40% (six patients) and in 28 days was 66.6% (10 patients). Median hematoma volume was 10.53 ml (IQR: 5.42 to 31.75) and hemoventricle volume was 8.86 (IQR: 0 to 27.08). Plasmatic iron concentration was higher in nonsurvivors than in survivors (496.04×58.5 mg/dl, *P *= 0.05) 24 hours after the event. CSF cytometry and lymphocyte count was more increased in nonsurvivors than in survivors (WBC count: 247.5 × 3 cells/ ml, *P *= 0.01 and lymphocyte count: 179×5 cells/ml, *P *= 0.01) 24 hours after the event. Plasmatic IL-2, IL-6, IL-8, and IL-12 levels are higher in nonsurvivors than in survivors. CSF IL-4 levels were higher in survivors and CSF IL-17 levels were higher in nonsurvivors than in survivors. There was no correlation between plasmatic or CSF levels of enolase and S100-B with mortality. Plasmatic hemopexine and haptoglobin levels also do not seem to be associated with survival.

## Conclusion

A systemic and local proinflammatory response is associated with higher mortality in patients with hemorrhagic stroke. CSF IL-4 higher concentrations are associated with better prognosis.

